# Supplementary material for: The interactome of intact mitochondria by cross-linking mass spectrometry provides evidence for coexisting respiratory supercomplexes
Source: Mol Cell Proteomics. 2017 Dec 8;17(2):216–32. doi: 10.1074/mcp.RA117.000470 (PMC5795388; doi:10.1074/mcp.RA117.000470)
Supplement: Supplemental Data [file supp_RA117.000470_133922_0_supp_23978_fzffwf.zip › spectra_annotation/mito_DR_spectra_annotation/493-2-4-1-9-1.pdf]

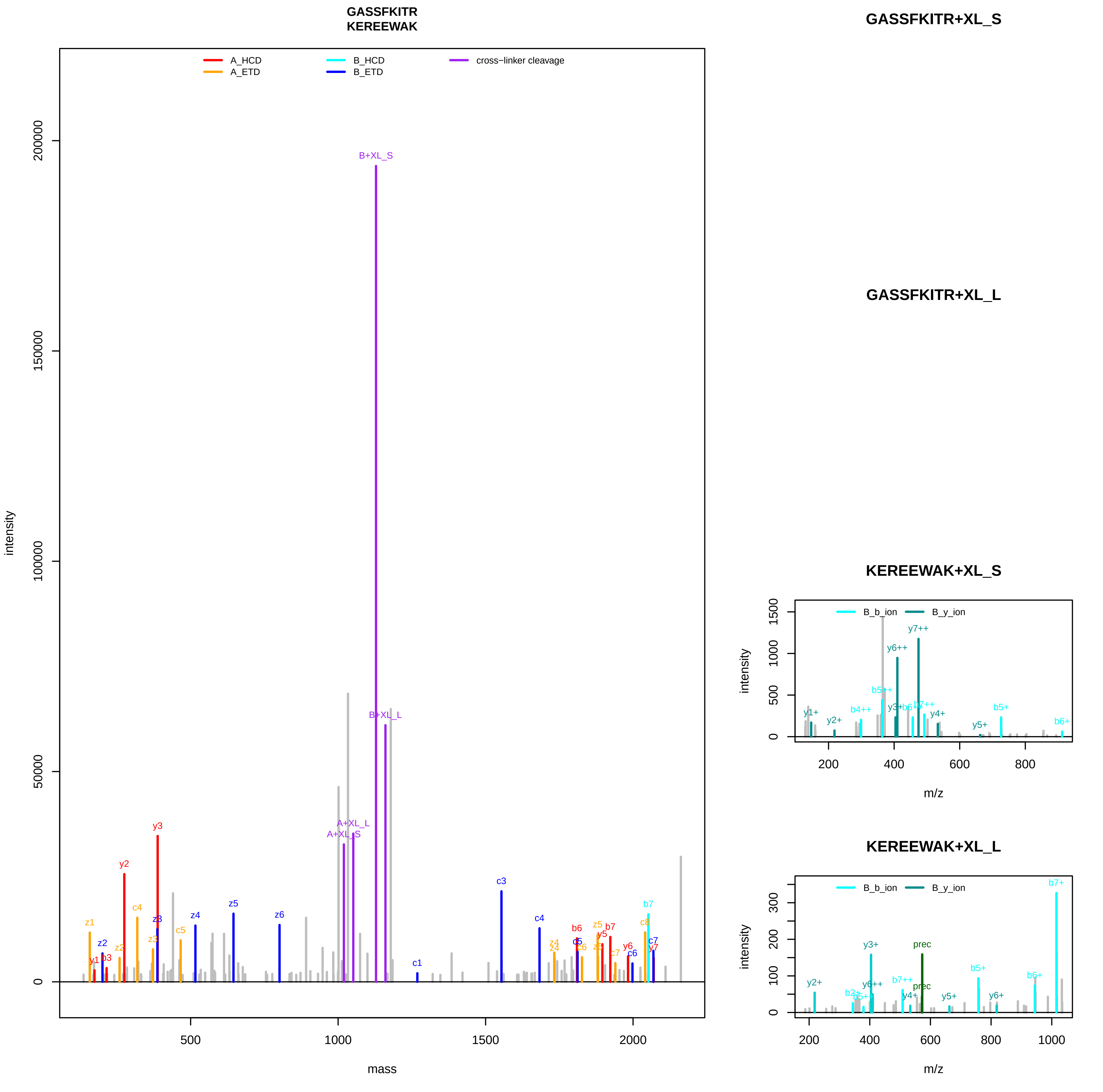

GASSFKITR
KEREEWAK
A_HCD
A_ETD
B_HCD
B_ETD
cross−linker cleavage
B+XL_S
B+XL_L
A+XL_L
A+XL_S
intensity
mass
0
50000
100000
150000
200000
500
1000
1500
2000
y1 b3 y2 y3 z1 z2 z3 z4 z5 z6 c1 c3 c4 c5 c6 c7 c8 b6 b7 y5 y6 y7 b7
GASSFKITR+XL_S
GASSFKITR+XL_L
KEREEWAK+XL_S
KEREEWAK+XL_L
B_b_ion
B_y_ion
m/z
y1+ y2+ b4++ b5++ y6++ y7++ y3+ b6++ b7++ y4+ y5+ b5+ b6+
y2+ b2+ y3+ y6++ b7++ prec y4+ y5+ b5+ y6+ b6+ b7+
